# Global Examination of Mental State: An open tool for the brief evaluation of cognition

**DOI:** 10.1002/brb3.2710

**Published:** 2022-07-21

**Authors:** Sara Mondini, Sonia Montemurro, Veronica Pucci, Adele Ravelli, Matteo Signorini, Giorgio Arcara

**Affiliations:** ^1^ Department of Philosophy, Sociology, Education and Applied Psychology (FISSPA) University of Padua Padua Italy; ^2^ Human Inspired Technology Research Centre University of Padua Padua Italy; ^3^ IRCCS San Camillo Hospital Venice Italy; ^4^ Gruppo Veneto di Diagnostica e Riabilitazione (GVDR) Padua Italy

**Keywords:** cognitive screening, neuropsychology, open tool, psychometric properties

## Abstract

**Background:**

The aim of this paper is to present a freely accessible new instrument for the evaluation of cognition: the Global Examination of Mental State (GEMS).

**Methods:**

It is made up of 11 items tapping into a range of skills, such as Orientation in time and space, Memory, Working memory, Visuo‐spatial, Visuo‐constructional and Planning abilities, Perceptual and visual Attention, Language (Naming, Comprehension, and Verbal fluency), and Pragmatics.

**Results:**

The psychometric strengths of this screening are: (1) extensive and updated normative data on the adult Italian population (from 18 to 100 years old); (2) absence of ceiling effect in healthy individuals, which allows to better detect interindividual variability; (3) comparison of the global scores with normative data taking into account Cognitive Reserve rather than only education, thus increasing diagnostic accuracy; (4) thresholds for significant change over time and the possibility to use parallel versions (GEMS‐A/GEMS‐B) for test‐retest; (5) solid psychometric properties and data on discriminant validity; and (6) free access to all materials (record forms, instructions, and cut‐off scores) on the web under a Creative Common License.

**Conclusions:**

With all these characteristics, GEMS could be a very useful paper‐and‐pencil instrument for cognitive screening.

## Graphical abstract

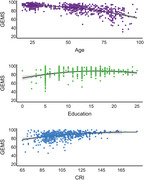

## 1 INTRODUCTION

Screening tests aim to rapidly and accurately identify the cognitive status of a person and are essential for both clinical purposes and research. In many health services, knowing the “global” cognitive status of a patient can be extremely useful for diagnostic and prognostic purposes (Morley et al., 2015). Cognitive deficits may be the sign of a primary neurological disorder, but they may also be the result of indirect effects of other pathologies on brain functioning. In these cases, a concise picture of a patient's cognitive functioning can be useful in order to better define the diagnosis or to tailor the treatment. However, as an extensive neuropsychological evaluation is not always necessary or possible, short cognitive screenings are often an optimal choice (Rodrigues et al., 2019; Roebuck‐Spencer et al., 2017) in research and clinical services (Lezak et al., 2012; Malloy et al., 1997) and in many cases to monitor a treatment or the evolution of a pathology. An example of pathologies with possible indirect effects on cognition are *cardiovascular diseases* (Muller et al., 2007); *hypertension* (Muela et al., 2017); *metabolic disorders* (Feinkohl et al., 2019; Zilliox et al., 2016)*; obesity* (Buie et al., 2019); *obstructive sleep apnea syndrome* (Devita et al., 2017); and *eating disorders* (Grau et al., 2019). These are examples of cases in which a brief and general description of patients’ cognitive functioning is of greater advantage than a detailed evaluation (Zangrossi et al., 2021).

Another reason to prefer screenings is the fact that patients might be unable to cope with the demands of a whole neuropsychological assessment, which may last very long and sometimes require a high amount of resources (Plass et al., 2010).

Moreover, screenings may provide a concise picture of cognition, rather than a patchy frame of different cognitive functions (Riello et al., 2021). In many cases, for example, it is more useful to identify the general cognitive functioning of a patient rather than their specific disorders in order to evaluate day‐to‐day capacities (Block et al., 2017).

Furthermore, screenings are useful in many fields of research especially to verify inclusion/exclusion criteria or to evaluate the effect of experimental variables on cognition.

However, a limitation of screenings is that, although commonly used with different pathologies, they are developed for specific clinical populations. For example, Mini‐Mental State Examination (Folstein et al., 1975) (MMSE) as well as Addenbrooke's Cognitive Examination‐Revised (Mioshi et al., 2006) were originally focused on Alzheimer's type dementia; Montreal Cognitive Assessment (Nasreddine et al., 2005) was developed specifically to detect mild cognitive impairment (MCI, the predementia stage); Oxford Cognitive Screen (Demeyere et al., 2015) is tuned specifically for stroke disorders; Edinburgh Cognitive and Behavioural ALS Screen (Abrahams et al., 2014) considers patients with amyotrophic lateral sclerosis; Brief Assessment of Cognition in Schizophrenia (Keefe et al., 2004) applies to patients with schizophrenia; and Rao's Brief Repeatable Battery (Rao et al., 1991) to patients with multiple sclerosis.

On the contrary, other screenings are suitable for a wide range of pathologies, but determine only specific deficits; for example, the Frontal Assessment focuses on executive dysfunctions.

A common issue of screening tests is that their global score is derived from adding up all the items of each subtask, but not yielding an equal weight on the global score for all cognitive subtasks. For example, in MMSE, the total score is 30, where 10 points (one third) are allotted to orientation (in space and time), whereas only 1 point is for constructional apraxia. Another example is in the Montreal Cognitive Assessment (MoCA): 6 points are attributed to the Visuospatial/Executive section, while 3 points are attributed to the Naming section.

Moreover, screening tests sometimes lack data on important psychometric properties, which limit their potential as assessment tools. A recent systematic review on Italian tests has shown that validity and reliability are often neglected properties, while thresholds for significant change are hardly ever reported (Aiello et al., 2022).

Another limitation is that they are often used to draw general inferences on cognition even if there is no validation in this sense: often, there are no data showing that the screenings correlate satisfactorily with wider batteries or with general tests of cognition.

Another important point is that scores of screenings, as is the case for all cognitive tests, have so far only been adjusted for age, education, and sex (Strauss et al., 2006), although other variables may be at play and influence cognition (e.g., sociobehavioral or socioeconomic factors Fratiglioni et al., 2004; Livingston et al., 2017; Mondini et al., 2022; Ward et al., 2015). From this perspective, education is only one component of the concept of Cognitive Reserve (CR, Stern, 2003), which is well recognized as a comprehensive measure of abilities and knowledge acquired during life (Stern & Barulli, 2009), and as a modulator of cognitive performance (Lojo‐Seoane et al., 2018; Mitchell et al., 2012; Montemurro et al., 2019; Steffener & Stern, 2012). CR is considered a protective factor against major neurocognitive decline (Mondini et al., 2022), whereas in other pathologies, CR shows a positive effect on recovery after brain damage (Hindle et al., 2014; Menardi et al., 2020; Nunnari et al., 2014). Thus, in order to have a clearer picture of the examinees’ performance, test scores should be better adjusted for CR rather than merely age and education.

Finally, following Open Science principles and sharing tools with appropriate licenses (as a Creative Common) could be a way to share neuropsychological instruments with professionals and researchers.

With this in mind, we present a new screening test, the Global Examination of Mental State (GEMS), which provides a fast measure of global cognition in approximately 10 min. This screening is easy to administer and takes into account many psychometric and methodological aspects often neglected in screening tests.

## 2 MATERIALS AND METHODS

### 2.1 Participants

Healthy Italian volunteers (635; 396 females) were recruited in different social groups, organizations, and in other environments without connections with clinical settings. Inclusion criteria were: age over 18, Italian mother tongue, and autonomy in principal daily living activities. Persons with neurological or psychiatric diseases were excluded.

Mean age of the whole sample was 51.8 (SD = 21.57) ranging from 18 to 98 and mean education was 12.85 (SD = 4.91) ranging from 0 to 25 years (one participant was illiterate, but able to carry out the test). All participants underwent the administration of the Cognitive Reserve Index questionnaire (CRIq) in its digital form (average CRIq = 100, SD = 16.84, range: 65–181).

GEMS and CRIq were also administered to 49 patients with Parkinson's disease (16 females) recruited in the *Gruppo Veneto di Diagnostica e Riabilitazione* (GVDR, Padua, Italy). Mean age was 73.7 (range 44–85; SD 8.97), mean education was 9.99 (range 5–18; SD = 4.13), and mean CRIq was 107.4 (range 73–157; SD = 18.2). These patients were also administered MMSE (Folstein et al., 1975) and the MoCA (Nasreddine et al., 2005). Their mean score at MoCA was 23/30 (SD = 0.97; range 7–29); at MMSE was 26/30 (SD = 5.26; range 8–30); and at GEMS was 66.9/100 (SD = 18.4; range 21.8–96.8).

All participants took part voluntarily in this study, signed the informed consent, were aware they could stop and withdraw from the testing at any time. This study was approved by the Ethical Committee of the School of Psychology, University of Padua and it adheres to the Declaration of Helsinki.

All participants were administered: (1) GEMS (in two parallel forms GEMS‐A and GEMS‐B) for normative data collection and (2) CRIq (Nucci et al., 2012) for the measurement of CR. Two subgroups from the whole sample underwent further tests: (3) MoCA (Nasreddine et al., 2005) and (4) ENB‐2 (Esame Neuropsicologico Breve, Brief Neuropsychological Examination, Mondini et al., 2011) to verify construct validity.

### 2.2 Study design

**FIGURE 1:**
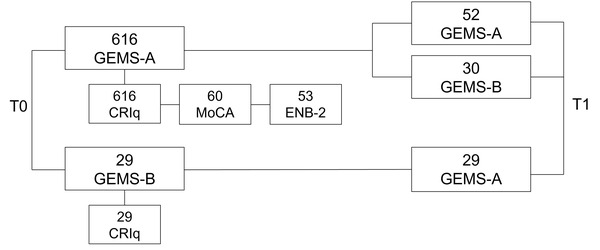
The figure describes the data collection design of GEMS. Blocks on the right part of the figure are the data collected at T0, while blocks on the right side are data collected at T1 (1–3 months after T0). The label in each block refers to the test/questionnaire administered. The number above each test indicates the number of participants data were collected from

GEMS‐A and CRIq were administered to 616 participants, while another 29 were tested with GEMS‐B and CRIq. From the 616 group, 60 participants also underwent the MOCA (Nasreddine et al., 2005), and 50 the ENB‐2 (Mondini et al., 2011). The assignment to one of these three groups was random. After 1–3 months, 52 individuals underwent GEMS‐A, while 59 were retested with GEMS‐B. See Figure 1 for more details about the data collection design.

### 2.2 Materials

GEMS is made up of three sheets to complete and a fourth with four colored pictures to name. The subtasks are listed in a definite order. GEMS begins by collecting examinees’ personal data and their cognitive reserve (CRIq, Nucci et al., 2012).

GEMS is composed of 11 subtasks assessing: Orientation; Immediate Memory; Months Backward; Puzzle; Clock; Delayed Memory; Picture Naming; Verbal Comprehension; Visual Attention; Verbal Fluency; and Metaphor Comprehension.

Each of the 11 items gives a raw score, which is then proportionally recorded in a way that each item (representing mainly one cognitive function) weighs as any other on the final score. The total score aims to represent a cognitive profile without a priori relevance in any functions. Thus, GEMS does not address any specific diagnosis or disorder.

GEMS‐A and GEMS‐B are freely available on the web at the OSF link: https://osf.io/4t5a8 with instructions and an Excel file to convert the raw scores and cut‐offs.

The Cognitive Reserve Index questionnaire (Nucci et al., 2012, CRIq, available on https://www.cognitivereserveindex.org/NewEdition/index.html) is a semistructured interview to measure a person's CR considering education, working activity, and leisure time activities during the lifespan.

The MOCA (Nasreddine et al., 2005) is a screening test, which takes 10 min to be administered and assesses several cognitive domains.

The ENB‐2 (Mondini et al., 2011, Brief Neuropsychological examination) is a battery of 14 tests (Digit Span; Story Recall, Immediate and Delayed; Interference Memory, 10 and 30 s; Trail Making Test part A and B; Token; Phonemic Fluency; Abstract Reasoning; Cognitive Estimation; Overlapping Figure; Spontaneous Drawing; Copy Drawing; Clock Drawing; and Apraxia) with a total final score.

### 2.3 Statistical analysis

An initial item analysis aimed to identify and select satisfactory tasks and items of GEMS. The items targeting verbal functions were selected according to psycholinguistic properties like frequency and lexical agreement.

Construct validity (with MoCA and ENB‐2) was assessed by Pearson's correlations. Internal consistency was calculated through a standardized Cronbach's alpha. Test‐retest and parallel‐form reliability were analyzed through Pearson's correlations. We also calculated significant change thresholds by means of a regression‐based approach (Crawford & Garthwaite, 2007).

We assessed the relationship of the variables age, sex, education, and CRI (as a proxy of CR) with GEMS, by a series of multiple regressions, entering total GEMS score as dependent variable (see Supplementary Material 3). Based on the best fitting model, clinical cut‐offs were obtained following Crawford and Garthwaite's method (Crawford & Garthwaite, 2007). Discriminant validity was calculated by means of receiver operating characteristic (ROC) curve, to evaluate the area under the ROC curve (AUC) to discriminate between healthy individuals and those with Parkinson's disease. All analyses, except for the parallel‐form reliability, refer to GEMS‐A. The analyses were performed with R software (version 4.1.0; R Development Core Team, 2021).

## 3 RESULTS

The mean score of 635 GEMS total scores was 83.41/100 (SD = 12.8; range 21–99). The distribution was left‐skewed, with no ceiling effect (see Table 1 for descriptive statistics of each item and total scores).

**TABLE 1:** Descriptive statistics of the demographic features of the sample and their scores for each GEMS task

	Mean	SD	Median	Min	Max	Kurtosis	Skewness	Q1	Q3
Age	51.85	21.5	54	18	98	−1.2	0.04	28	69
Education	12.82	4.9	13	0	25	−0.66	−0.14	8	17
CRIq	103.8	16.9	100	65	181	1.03	0.83	92	114
Orientation	8.79	0.8	9	2	9	34.78	−5.26	9	9
Immediate Memory	6.76	1.9	8	0	9	−0.68	−0.64	6	8
Months Backwards	8.07	2	9	0	9	5.48	−2.42	9	9
Puzzle	8.11	2.4	9	0	9	4.99	−2.55	9	9
Clock	7.9	2.4	9	0	9	3.88	−2.21	9	9
Delayed Memory	5.08	2.5	6	0	9	−0.76	−0.2	3	8
Picture Naming	8.44	1.2	9	4	9	5.23	−2.33	9	9
Verbal Comprehension	8.57	1.6	9	0	9	12.91	−3.71	9	9
Visual Attention	8.05	1.7	9	0	9	5.2	−2.13	7	9
Verbal Fluency	5.57	2.1	6	0	9	−0.5	−0.17	4	7
Metaphor Comprehension	8.12	2.7	9	0	9	5.3	−2.7	9	9
GEMS_total	83.45	12.8	87.5	21	100	3.9	−1.81	79	92

### 3.1 Internal consistency

Results showed high internal consistency (alpha = 0.81) and each item showed a high correlation with the global score: 0.80 for Orientation, 0.79 for Immediate Memory, 0.79 for Backward Months, 0.80 for Puzzle, 0.77 for Clock, 0.78 for Delayed Memory, 0.79 for Naming, 0.80 for Comprehension, 0.81 for Attention, 0.78 for Verbal Fluency, and 0.80 for Metaphor Comprehension.

### 3.2 Construct validity

In order to verify GEMS capacity to measure global cognition, a subsample of 60 participants were further assessed with MoCA and ENB‐2. GEMS correlated with MoCA (*r* = 0.723, *p* < .001) and with ENB‐2 (*r* = 0.811, *p *< .001). To corroborate these results, we performed two additional regressions with GEMS total scores as dependent variable: in the first one, MoCA was the predictor (Adj. *R* ^2^ = 0.514, *p *< .001), while in the second, ENB‐2 was the predictor (Adj. *R* ^2^ = 0.651, *p *< .001). Both models strengthened a satisfactory construct validity.

### 3.3 Test‐retest reliability, practice effect, and parallel forms

GEMS test‐retest reliability measured on 52 participants was very good (test‐retest: *r* = 0.845, *p* < .001) ranging from good to excellent for each task, except for Verbal Comprehension. Table 2 shows more details.

**TABLE 2:** The table shows the values of test‐retest reliability (Pearson's *r*) and practice effect (paired *t*‐tests) of scores of single tasks and global score after the administration of GEMS‐A followed by a second administration of GEMS‐A (GEMS A‐A) compared with the administration of GEMS‐A followed by the administration of GEMS‐B (GEMS A‐B)

	Test‐retest reliability(Pearson's *r*)		Mean difference(Retest minus)		Practice effect(paired *t‐test*)	
GEMS tasks and total score	GEMSA‐A	GEMSA‐B	GEMSA‐A	GEMSA‐B	GEMSA‐A (df = 51)	GEMSA‐B (df = 58)
Orientation	1	–0.069	+0.15	+0.48	*t* = 2.06, *p* = .044	*t* = 3.35, *p* = .001
Immediate Memory	0.600	0.625	+0.46	+0.25	*t* = 3.91, *p* < .001	*t* = 1.40, *p* = .168
Months Backwards	0.392	0.493	+0.09	+0.07	*t* = 0.87, *p* = .389	*t* = −0.38, *p* = .709
Puzzle	0.160	0.516	–0.01	+0.12	*t* = −0.15, *p* = .877	*t* = 0.42, *p* = .678
Clock	0.775	0.573	–0.12	+0.11	*t* = −0.63, *p* = .532	*t* = −0.44, *p* = .659
Delayed Memory	0.741	0.709	+0.04	+0.43	*t* = 0.26, *p* = .792	*t* = 1.67, *p* = .101
Naming	0.544	0.249	+0.25	+0.38	*t* = 2.09, *p* = .041	*t* = 2.26, *p* = .028
Verbal Comprehension	0.035	1	0	0	*t* = 0, *p* = 1	*t* = 0, *p* = 1
Visual Attention	0.484	0.146	–0.13	–0.09	*t* = −1.22, *p* = .226	*t* = −0.49, *p* = .623
Fluency	0.766	0.585	+1.46	–0.07	*t* = +3.45, *p* < .001	*t* = −0.32, *p* = .749
Metaphor	0.485	0.296	+0.52	–0.16	*t* = +1.77, *p* = .083	*t* = −0.37, *p* = .709
GEMS total	0.845	0.774	+2.4	+1.16	*t* = −3.33, *p* = .001	*t* = 1.42, *p* = .162

Practice effect was calculated with a series of paired *t*‐tests: a significant practice effect was found in 4 out of 11 tasks (i.e., Orientation, Immediate Memory, Naming, and Fluency) and in the GEMS total scores.

To account for practice effect, GEMS (hence GEMS‐A) was paired with a second form: GEMS‐B. A subsample of 59 participants was assessed with both versions and results showed good correlation (Pearson's *r *= 0.774, *p* < .001). The correlation coefficients deriving from GEMS‐A/GEMS‐A and GEMS‐A/GEMS‐B were verified using the Fisher *r*‐to‐*z* transformation, and results showed no significant differences (two‐tailed *p *= .289).

Practice effect between GEMS‐A and GEMS‐B calculated with paired *t*‐tests showed no practice effect (Table 2).

### 3.4 Threshold for significant change

A regression‐based approach was used to calculate significant (Crawford & Garthwaite, 2007) between two measurements, which allows to predict the second score from the first. If the observed score at the second measurement is significantly “far” from the predicted one, then a significant change may have occurred. Significant change values are provided in the Supplementary Materials (Supplementary Materials 2). Thresholds for significant change are available for using GEMS‐A followed by GEMS‐A or GEMS‐B.

### 3.5 Effect of demographic variables

The effect of age, sex, education, and CRI was assessed in multiple regressions to derive clinical cut‐offs (see below). Results show that age, education, and CRI are significant predictors of GEMS, whereas sex has no effect. In particular, age has a negative relationship with GEMS, whereas education and CRI have a positive one. Interestingly, the main effect of CRI was stronger than that of education.

**FIGURE 2:**
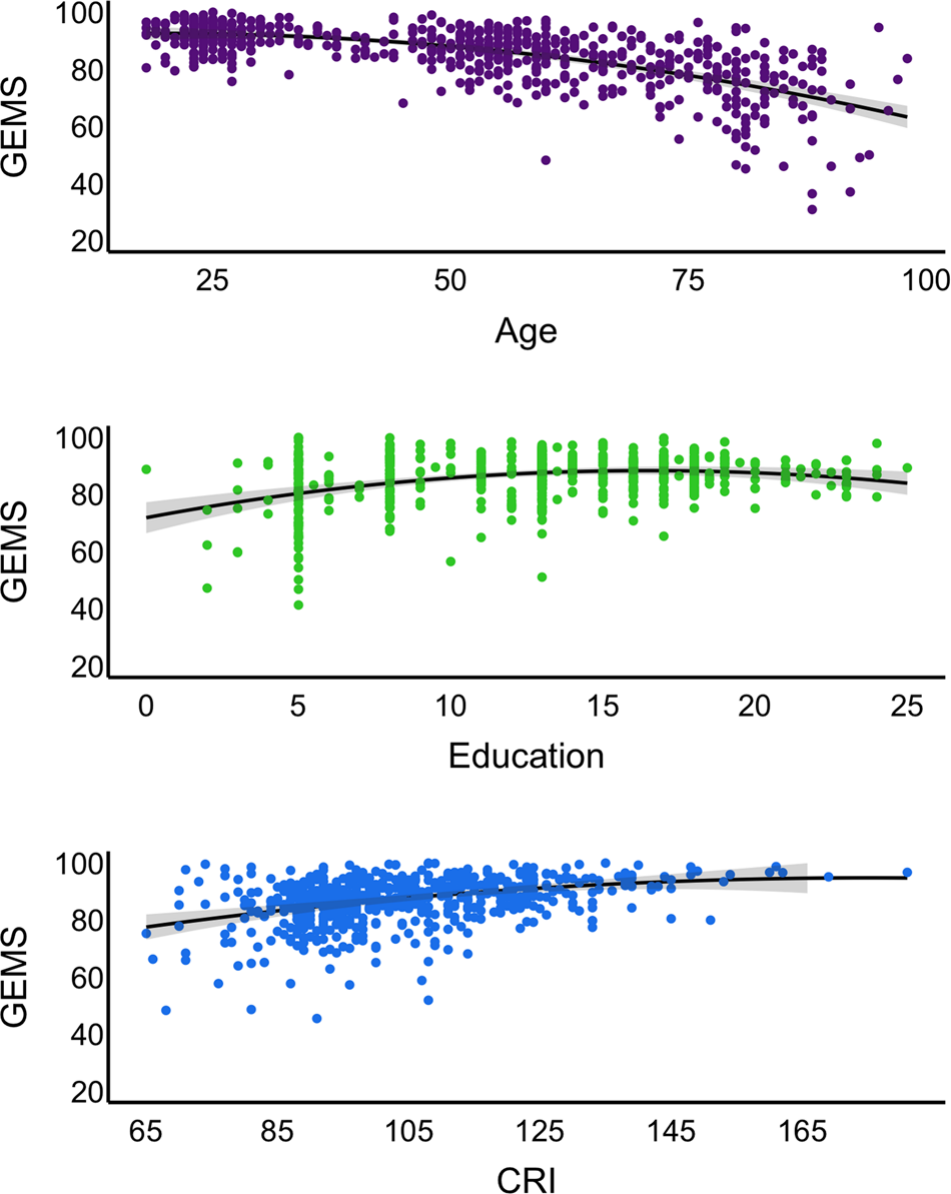
Effect of age, education, and cognitive reserve on GEMS scores. Age, education, and CRI are reported on the x‐axis, and GEMS scores are reported on the y‐axis. Quadratic terms of age, education, and CRI are included into the regression models

Visual inspection of the partial residuals indicated that age, education, and CRI were nonlinearly related with GEMS. Models 4–6 were thus built to check whether including nonlinear terms would improve the model fit. We used the modified version of the Akaike Information Criterion for model comparison and Adjusted *R* ^2^ to select the best fitting model. Model 6, including age, sex, and CRI, and the quadratic terms for age (age2), education (education2), and CRI (CRI2) showed the minimum loss of information (Burnham et al., 2011) and it was used for generating cut‐offs. For more details, see Figure 2 and Table S3.

### 3.6 Cut‐offs

Cut‐offs were calculated using the Crawford and Garthwaite's (Crawford & Garthwaite, 2007) approach. Participant score is predicted from demographic variables (age, sex, education, and CRI) using the Model 6 reported in Table S3. An important feature of this method is that it takes into account the problem of the estimate for extreme values of the predictors, and it is specifically designed to compare a single case to a control group. Cut‐off tables of GEMS are reported in Table S4 with a few of the combinations of age, sex, education, and CRI. The precise cut‐offs for all possible combinations of sociodemographic variables (age, sex, education, and CRI) can be calculated by using a Shiny App available on the OSF.

### 3.7 Discriminant validity

**FIGURE 3:**
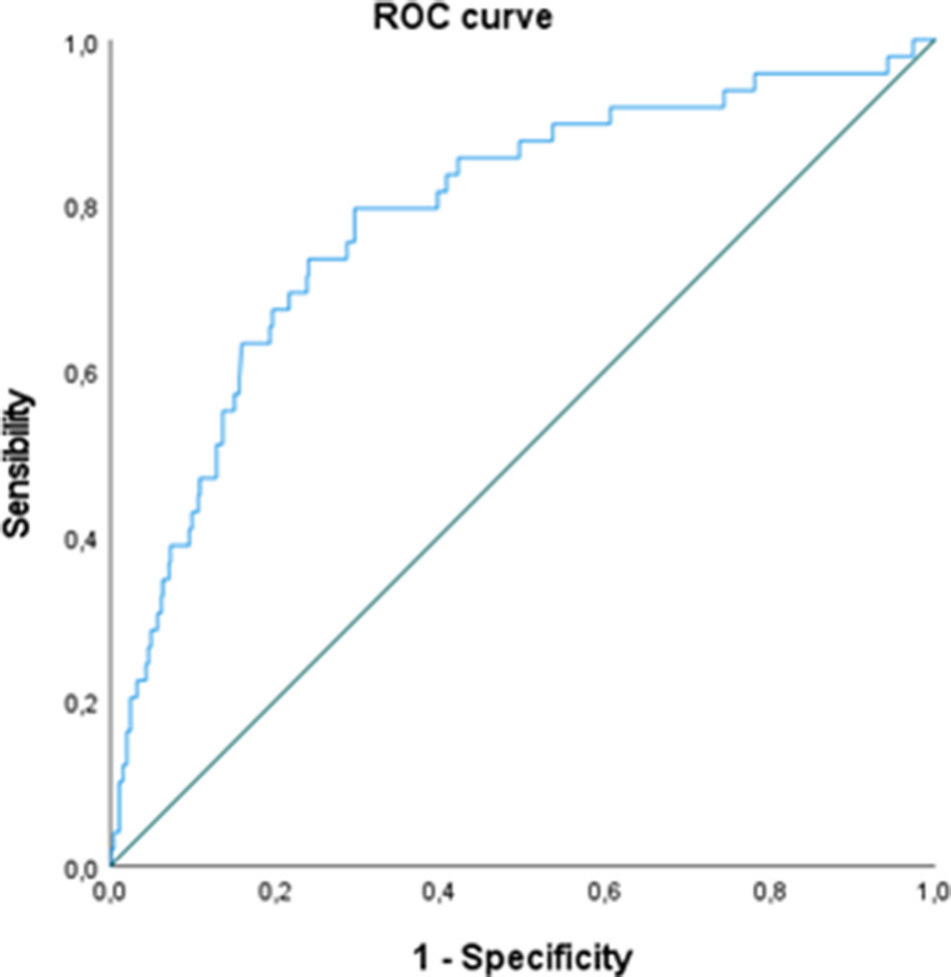
ROC curve for GEMS scores in discriminating between healthy participants and patients with Parkinson's disease

GEMS was administered to 49 patients with Parkinson's disease. The AUC of the model was 0.786 (SE = 0.036, 95% CI, 0.716, 0.856), showing a very good discriminant validity (Figure 3).

## 4 DISCUSSION

In this work, we have presented GEMS, a new paper‐and‐pencil screening test to investigate global cognition and impairments of any origin/etiology.

GEMS psychometric properties, normative data, and cut‐offs based on a well‐represented sample for the current Italian population are reported. The score of the 11 GEMS tasks was obtained by transforming the raw scores into proportions and then averaged, so that each task contributes with equal weight to the final composite score which ranges from 0 to 100 (see the same approach in Arcara & Bambini, 2016).

GEMS showed a high internal consistency, indicating that each task has a high correlation with the global score. Furthermore, GEMS showed optimal correlation with a complete and extensive neuropsychological battery (ENB‐2 Mondini et al., 2011) and with a well‐known cognitive screening (MoCA, Nasreddine et al., 2005). This highlights the potential of GEMS to measure the underlying construct (good convergent validity). Test‐retest reliability (GEMS‐A and GEMS‐A) was optimal, demonstrating score stability across repeated measurements. A practice effect was also found, but this was reduced using the parallel version GEMS‐B. Indeed, the two versions showed high correlation and no practice effect. Thresholds of significant changes are also reported, allowing to detect a significant improvement/decrement over time. Although parallel forms can certainly reduce practice effect, a significant change approach to detect possible meaningful changes over time is important to monitor cognition (see Aiello et al., 2022 for further considerations on the psychometric properties of cognitive screening).

Younger individuals performed better than older ones and those with higher education and/or higher CR performed better than those with lower education and lower CR; no sex difference was found.

GEMS cut‐offs are generated considering not only age and education but the more comprehensive score of cognitive reserve, which provides a more precise expectation on performance and better understanding of the possible evolution of the profile. Indeed, we found that cognitive reserve is a more reliable predictor of cognitive performance than education (for similar results, see Montemurro et al., 2021).

Comparison with a clinical population showed that GEMS has high sensitivity and high specificity in discriminating healthy individuals from individuals with Parkinson's disease.

In addition to its psychometric properties, this cognitive screening has other strengths.

In the spirit of Open Science, GEMS record forms, instructions, and cut‐off scores are freely available on the web under a Creative Common license and interested neuropsychologists can use them in different clinical or research settings. Furthermore, the accessibility to all the materials will allow authors from different countries to easily translate and adapt GEMS into specific cultures and languages and proceed with the collection of normative data in different populations.

GEMS is not exempt from limitations. For example, information about inter‐rater reliability could not be collected due to data being gathered during the recent pandemic restrictions. Furthermore, discriminating validity was measured with Parkinson's patients, but other clinical populations should be integrated or considered in future studies.

Despite the above limitations, and although the diagnostic capacity of any screening may not be comparable to a comprehensive test battery (Roebuck‐Spencer et al., 2017), GEMS could significantly contribute to and enhance the quality of the neuropsychologist's toolkit.

## FUNDING

Article payment supported by Ricerca Corrente funds of the Italian Ministery of Health to IRCCS San Camillo Hospital. GA and SM were supported by the Italian Ministry of Health. SM and VP were supported by D.O.R. (Dotazione Ordinaria ricerca) 2021 from University of Padua to SM.

### PEER REVIEW

The peer review history for this article is available at https://publons.com/publon/10.1002/brb3.2710

## Supporting information

Supplementary material

## Data Availability

The datasets generated during and/or analyzed during the current study are not publicly available due to information that could compromise participant privacy but are available from the corresponding author on reasonable request.
