# Supplementary material for: Global Examination of Mental State: An open tool for the brief evaluation of cognition
Source: Brain Behav. 2022 Jul 21;12(8):e2710. doi: 10.1002/brb3.2710 (PMC9392517; doi:10.1002/brb3.2710)
Supplement: Supplementary file 1 — Supplementary material [file BRB3-12-e2710-s001.docx]

**Global Examination of Mental State (GEMS):**

**An *open tool* for the brief evaluation of cognition**

Mondini S., Montemurro S., Pucci, V., Ravelli A., Signorini M., and Arcara G.

**Supplementary Materials**

**Supplementary Material - 1 Description of the GEMS sub-tasks**

GEMS is made up eleven tasks:

1) Orientation: to evaluate orientation in time and space, four questions are asked.

2) Immediate Memory: to evaluate memory six Italian words were selected: all bi-syllabic and phonologically dissimilar, with equivalent lexical frequency (from SUBLEX-IT(Crepaldi et al., 2015)), and with high semantic distance (from Word-Embeddings Italian Semantic Spaces(Marelli, 2017)).

3) Months Backward Test: to evaluate working memory the examinee is asked to list months backward starting from October and skipping one month at a time (e.g., December, October, August, and so on, up to five times).

4) Puzzle: to evaluate visuo-constructional abilities the examinee is asked to recognize a simple drawing of a train and re-assemble the figure divided in four parts within a grid.

5) Clock: to test visuo-constructional-planning abilities the examinee is asked to insert in the empty circle on the sheet all the numbers of a traditional clock and place the hands on a specific time. Correct collocation of the numbers, the correct positions of the hands, and their correct length are considered for the scoring.

6) Delayed Memory: to test the delayed verbal memory (after about 5 minutes from the presentation), the examinee is asked to recall the six words previously repeated in the Immediate Memory.

7) Picture Naming: to verify verbal lexical access the examinee has to name four non-living pictures balanced for lexical frequency, familiarity, age of acquisition, percentage of name agreement, and visual complexity, manipulability and typicality (values are taken from Navarrete et al.(Navarrete et al., 2019)).

8) Verbal Comprehension: to verify the verbal comprehension of a simple command, the examinee had to perform a three-step request.

9) Visual Attention: this is a barrage task of visually similar stimuli to evaluate visual-selective attention.

10) Verbal Fluency: to verify lexical access and executive functioning.

11) Metaphor Comprehension: to evaluate pragmatic abilities with figurative language the examinee has to read and recognize the meaning of a metaphoric.

**Supplementary Material 2 – Thresholds of significant changes**

The Table allows to verify if a significant change occurred, according to regression-based approach(Crawford & Garthwaite, 2007), after a second administration of GEMS-A in the same individual. The thresholds are available for both GEMS A and B.

To determine if in the second assessment a significant change may be occurred, first find the observed value at the first administration in the central column, then, check the row corresponding to this value in the Table. On the right there are the values (for both GEMS-A and GEMS-B) above which a significant improvement is inferred, on the left there are the values below which a significant worsening is inferred (for both GEMS-A and GEMS-B).

If the score at the second measurement is outside these thresholds, then a significant change is inferred. On the contrary, if the observed value falls within these thresholds (threshold values included), no significant change is inferred.

According to this approach, if no change is occurred at second measurement, less than 5% of the population will exceed the upper thresholds and less than 5% of the population will fall below the lower thresholds.

Gray cell values are not observed on the sample but just inferred, use them with caution. In some cases the thresholds could not be calculated (for example if the predicted value exceeded the range of possible score values). In such cases a hyphen is reported.

| Lower threshold with GEMS-B | Lower threshold with GEMS-A | **Observed value 1st administration**  **GEMS-A** | Upper threshold with GEMS-A | Upper threshold with GEMS-B |
| --- | --- | --- | --- | --- |
| - | - | **0** | 31 | 39 |
| - | 1 | **2** | 33 | 40 |
| - | 2 | **4** | 34 | 41 |
| - | 4 | **6** | 36 | 43 |
| - | 6 | **8** | 37 | 44 |
| - | 8 | **10** | 39 | 45 |
| - | 10 | **12** | 40 | 46 |
| - | 12 | **14** | 42 | 48 |
| 15 | 14 | **16** | 43 | 49 |
| 17 | 16 | **18** | 45 | 50 |
| 19 | 18 | **20** | 46 | 51 |
| 21 | 20 | **22** | 48 | 53 |
| 22 | 22 | **24** | 49 | 54 |
| 24 | 23 | **26** | 51 | 55 |
| 26 | 25 | **28** | 52 | 56 |
| 27 | 27 | **30** | 54 | 58 |
| 29 | 29 | **32** | 55 | 59 |
| 31 | 31 | **34** | 57 | 60 |
| 33 | 33 | **36** | 58 | 62 |
| 34 | 35 | **38** | 60 | 63 |
| 36 | 37 | **40** | 61 | 64 |
| 38 | 38 | **42** | 63 | 66 |
| 39 | 40 | **44** | 64 | 67 |
| 41 | 42 | **46** | 66 | 68 |
| 43 | 44 | **48** | 67 | 70 |
| 44 | 46 | **50** | 69 | 71 |
| 46 | 48 | **52** | 70 | 72 |
| 48 | 49 | **54** | 72 | 74 |
| 49 | 51 | **56** | 73 | 75 |
| 51 | 53 | **58** | 75 | 76 |
| 53 | 55 | **60** | 77 | 78 |
| 54 | 57 | **62** | 78 | 79 |
| 56 | 58 | **64** | 80 | 80 |
| 58 | 60 | **66** | 81 | 82 |
| 59 | 62 | **68** | 83 | 83 |
| 61 | 64 | **70** | 85 | 85 |
| 62 | 65 | **72** | 86 | 86 |
| 64 | 67 | **74** | 88 | 88 |
| 65 | 69 | **76** | 90 | 89 |
| 67 | 71 | **78** | 91 | 90 |
| 69 | 72 | **80** | 93 | 92 |
| 70 | 74 | **82** | 95 | 93 |
| 72 | 76 | **84** | 96 | 95 |
| 73 | 77 | **86** | 98 | 96 |
| 75 | 79 | **88** | 99 | 98 |
| 76 | 81 | **90** | - | 99 |
| 78 | 82 | **92** | - | - |
| 79 | 84 | **94** | - | - |
| 81 | 86 | **96** | - | - |
| 82 | 87 | **98** | - | - |
| 83 | 89 | **100** | - | - |

**Supplementary Material Table 3 - Regression Models with demographic variables as predictors of interest and GEMS total score as dependent variable.**

The relationship of the variables age, sex, education, and CRI (as a proxy of CR) with GEMS was assessed troughth a series of multiple regressions, with total GEMS score as dependent variable. Age, education and CRI were included in multiple regressions as continuous predictors, whereas sex was included as a factorial variable. The best model was diagnosed (e.g., for possible outliers) and also visually inspected: following the procedure already used in Arcara and Bambini(Arcara & Bambini, 2016) and in Montemurro et al.(Montemurro et al., n.d.), we explored the possibility of improving the fit by allowing non-linear terms: for all variables that showed a non-linear trend, we tested whether adding quadratic terms yielded better models. Then, the AICc of all the models were compared and the one with the lowest AICc was chosen. Clinical cut-offs were obtained through the regression method of Crawford and Garthwaite(Crawford & Garthwaite, 2007) based on the best fitting model.

The Table reports the model name (first column); the intercept and the predictors entered in the regression model (second column); standard estimate and and standard error within brackets (third column); t-value associated with the model (fourth column); p-value associated with the model in which asterisks indicate p-value minor of 0.05 (fifth column); adjusted R2 associated with the model (sixth column); modified version of the Akaike Information Criterion values (AICc, seventh column), in which the lowest the AICc value, the better the model fit; Akaike Weights associated with the model (AICw, eighth column): probability of a model to be the best among the given set of models.

| **Regression Models – dependent variable: GEMS score** | | | | | | | |
| --- | --- | --- | --- | --- | --- | --- | --- |
| Model | Predictor | Standard Estimate | t-value | p-value | Adj. R2 | AICc (df) | AICw |
| Model 1 | Intercept | 103.32 (1.09) | 94.94 | <0.001* | 0.392 | 4794 (4) | 0.000 |
|  | Age | -0.37 (0.02) | -19.91 | <0.001* |  |  |  |
|  | Sex | -1.43 (0.81) | -1.75 | 0.080 |  |  |  |
| Model 2 | Intercept | 83.26 (1.93) | 43.11 | <0.001* | 0.504 | 4664 (5) | 0.000 |
|  | Age | -0.24 (0.02) | -11.86 | <0.001* |  |  |  |
|  | Sex | -1.58 (0.74) | -2.15 | 0.031* |  |  |  |
|  | Education | 1.04 (0.09) | 12.06 | <0.001* |  |  |  |
| Model 3 | Intercept | 71.40 (2.17) | 32.78 | <0.001* | 0.568 | 4548 (6) | 0.000 |
|  | Age | -0.38 (0.02) | -15.96 | <0.001* |  |  |  |
|  | Sex | -0.52 (0.69) | -0.75 | 0.452 |  |  |  |
|  | Education | 0.25 (0.11) | 2.19 | 0.028* |  |  |  |
|  | CRI | 0.28 (0.02) | 9.76 | <0.001* |  |  |  |
| Model 4 | Intercept | 64.56 (2.35) | 27.47 | <0.001* | 0.596 | 4508 (7) | 0.000 |
|  | Age | 0.24 (0.09) | 2.49 | 0.012* |  |  |  |
|  | Age^2 | -0.0 (0.00) | -6.59 | <0.001* |  |  |  |
|  | Sex | -0.49 (0.67) | -0.73 | 0.464 |  |  |  |
|  | Education | 0.34 (0.11) | 3.04 | 0.002* |  |  |  |
|  | CRI | 0.19 (0.03) | 6.37 | <0.001* |  |  |  |
| Model 5 | Intercept | 4.64 (2.99) | 18.26 | <0.001* | 0.612 | 4483 (8) | 0.349 |
|  | Age | 0.20 (0.09) | 2.15 | 0.003* |  |  |  |
|  | Age^2 | -0.0 (0.00) | -5.6 | <0.001* |  |  |  |
|  | Sex | -0.07 (0.66) | -0.11 | 0.908 |  |  |  |
|  | Education | 2.19 (0.37) | 5.87 | <0.001* |  |  |  |
|  | Education^2 | -0.06 (0.01) | -5.19 | <0.001* |  |  |  |
|  | CRI | 0.17 (0.03) | 5.76 | <0.001* |  |  |  |
| Model 6 | Intercept | 39.00 (9.14) | 4.26 | <0.001* | 0.612 | 4482 (9) | 0.651 |
|  | Age | 0.17 (0.09) | 1.78 | 0.075 |  |  |  |
|  | Age^2 | -0.0 (0.00) | -5.23 | <0.001* |  |  |  |
|  | Sex | -0.02 (0.66) | -0.03 | 0.971 |  |  |  |
|  | Education | 2.00 (0.38) | 5.16 | <0.001* |  |  |  |
|  | Education^2 | -0.06 (0.01) | -4.51 | <0.001* |  |  |  |
|  | CRI | 0.49 (0.17) | 2.75 | 0.005* |  |  |  |
|  | CRI^2 | -0.0 (0.00) | -1.80 | 0.070 |  |  |  |

**Supplementary Material Tables 4 - GEMS cut-offs**

The Supplementary Tables 3 report some possible cut-offs for few combinations of age, education, sex and CRI (Note: sex did not yield different cut-offs). The precise cut-offs for all possible combinations of socio-demographic variables (age, sex, education and CRI) can be calculated with the Shiny App available in the OSF website (<https://osf.io/4t5a8/>).

| Sex: M-F  CRI: 70-90 | | AGE | | | | | | | | | | | | | | |
| --- | --- | --- | --- | --- | --- | --- | --- | --- | --- | --- | --- | --- | --- | --- | --- | --- |
|  |  | **18-22** | **23-27** | **28-32** | **33-37** | **38-42** | **43-47** | **48-52** | **53-57** | **58-62** | **63-67** | **68-72** | **73-77** | **78-82** | **83-87** | **>88** |
| Education | **4-9** | 64 | 64 | 63 | 63 | 62 | 61 | 60 | 58 | 56 | 55 | 52 | 50 | 47 | 44 | 41 |
|  | **10-14** | 69 | 69 | 69 | 68 | 67 | 66 | 65 | 64 | 62 | 60 | 58 | 55 | 53 | 50 | 47 |
|  | **15-19** | 72 | 72 | 71 | 71 | 70 | 69 | 68 | 66 | 64 | 62 | 60 | 58 | 55 | 52 | 49 |
|  | **20-25** | 71 | 71 | 71 | 70 | 69 | 68 | 67 | 65 | 64 | 62 | 60 | 57 | 55 | 52 | 49 |

| Sex: M-F  CRI: 91-110 | | AGE | | | | | | | | | | | | | | |
| --- | --- | --- | --- | --- | --- | --- | --- | --- | --- | --- | --- | --- | --- | --- | --- | --- |
|  |  | **18-22** | **23-27** | **28-32** | **33-37** | **38-42** | **43-47** | **48-52** | **53-57** | **58-62** | **63-67** | **68-72** | **73-77** | **78-82** | **83-87** | **>88** |
| Education | **4-9** | 69 | 69 | 69 | 68 | 67 | 66 | 65 | 64 | 62 | 60 | 58 | 55 | 53 | 50 | 47 |
|  | **10-14** | 75 | 74 | 74 | 74 | 73 | 72 | 71 | 69 | 67 | 65 | 63 | 61 | 58 | 55 | 52 |
|  | **15-19** | 77 | 77 | 77 | 76 | 75 | 74 | 73 | 72 | 70 | 68 | 66 | 63 | 61 | 58 | 55 |
|  | **20-25** | 76 | 76 | 76 | 75 | 75 | 74 | 72 | 71 | 69 | 67 | 65 | 63 | 60 | 57 | 54 |

| Sex: M-F  CRI: 110-130 | | AGE | | | | | | | | | | | | | | |
| --- | --- | --- | --- | --- | --- | --- | --- | --- | --- | --- | --- | --- | --- | --- | --- | --- |
|  |  | **18-22** | **23-27** | **28-32** | **33-37** | **38-42** | **43-47** | **48-52** | **53-57** | **58-62** | **63-67** | **68-72** | **73-77** | **78-82** | **83-87** | **>88** |
| Education | **4-9** | 73 | 73 | 73 | 72 | 72 | 71 | 69 | 68 | 66 | 64 | 62 | 60 | 57 | 54 | 51 |
|  | **10-14** | 79 | 79 | 78 | 78 | 77 | 76 | 75 | 73 | 72 | 70 | 68 | 65 | 62 | 60 | 56 |
|  | **15-19** | 81 | 81 | 81 | 80 | 80 | 79 | 77 | 76 | 74 | 72 | 70 | 68 | 65 | 62 | 59 |
|  | **20-25** | 81 | 81 | 80 | 80 | 79 | 78 | 77 | 75 | 73 | 71 | 69 | 67 | 64 | 61 | 58 |

**References**

Arcara, G., & Bambini, V. (2016). A Test for the Assessment of Pragmatic Abilities and Cognitive Substrates (APACS): Normative Data and Psychometric Properties. *Frontiers in Psychology*, *7*, 70. https://doi.org/10.3389/fpsyg.2016.00070

Crawford, J. R., & Garthwaite, P. H. (2007). Using regression equations built from summary data in the neuropsychological assessment of the individual case. In *Neuropsychology* (Vol. 21, Issue 5, pp. 611–620). American Psychological Association. https://doi.org/10.1037/0894-4105.21.5.611

Crepaldi, D., Amenta, S., Mandera, P., Keuleers, E., & Brysbaert, M. (2015). SUBTLEX-IT. Subtitle-based word frequency estimates for Italian. *Annual Meeting of the Italian Association for Experimental Psychology, Rovereto*.

Marelli, M. (2017). Word-Embeddings Italian Semantic Spaces: A semantic model for psycholinguistic research. *Psihologija*, *50*(4), 503–520. https://doi.org/10.2298/PSI161208011M

Montemurro, S., Daini, R., Tagliabue, C., Guizzetti, S., Gualco, G., Mondini, S., & Arcara, G. (n.d.). Cognitive reserve estimated with a life experience questionnaire outperforms education in predicting performance on MoCA : Italian normative data. *Current Psychology*, 1–26.

Navarrete, E., Arcara, G., Mondini, S., & Penolazzi, B. (2019). Italian norms and naming latencies for 357 high quality color images. In *PLoS ONE* (Vol. 14, Issue 2). Public Library of Science. https://doi.org/10.1371/journal.pone.0209524
